# Prevalence, correlates and common conditions associated with adolescent dietary supplement use: a cross-sectional survey in Bangladesh

**DOI:** 10.1017/S1368980022002634

**Published:** 2023-06

**Authors:** Md. Bony Amin, Md. Aktarujjaman, Amatul Elah Meem, Ekhtear Hossain, Md. Nazrul Islam, Nitai Roy

**Affiliations:** 1 Faculty of Nutrition and Food Science, Patuakhali Science and Technology University, Patuakhali, Bangladesh; 2 Department of Biological Sciences and Chemistry, Southern University, A&M College, Baton Rouge, LA, USA; 3 Department of Post-Harvest Technology and Marketing, Faculty of Nutrition and Food Science, Patuakhali Science and Technology University, Patuakhali, Bangladesh; 4 Department of Biochemistry and Food Analysis, Faculty of Nutrition and Food Science, Patuakhali Science and Technology University, Patuakhali 8602, Bangladesh

**Keywords:** Dietary supplements, Adolescents, Prevalence and predictors, Bangladesh

## Abstract

**Objective:**

There is a broad spectrum of dietary supplements (DS) and their accessibility worldwide. However, little is known about the prevalence of DS use among Bangladeshi adolescents. This study estimates the prevalence, correlates and common conditions related to DS use.

**Design:**

A cross-sectional, convenient sampling strategy was adopted using an interviewer-administered, structured questionnaire.

**Setting:**

Kurigram and Patuakhali districts of Bangladesh.

**Participants:**

702 adolescents aged 10–19 years.

**Results:**

The overall prevalence of DS use was 83 %. The majority of participants (93·4 %) agreed that DS were good for health, and 28·3 % reported general health and well-being as the reason for using DS. The most frequently used supplements were multivitamins (38·6 %) and Ca (37 %). DS use was more common among adolescents who had ≤5 siblings, good health status, no chronic diseases, a positive impression that DS are good for health and who had the tendency to encourage DS to others. DS use was also higher among those who received DS information from healthcare providers, professional literature, friends, family and relatives.

**Conclusions:**

The prevalence of DS use is relatively higher among Bangladeshi adolescents compared to Bangladeshi adults and adolescents from other countries, highlighting the inclination towards DS use. Guidelines for safe DS use for adolescents are warranted to control DS use and prevent adverse effects.

An increasing rate in the era of using dietary or nutritional supplements is seen worldwide. In addition, adolescents’ interest in taking dietary supplements (DS) is countably growing, ranging from approximately 10 % to as high as 74 %^(1)^. There is no one particular reason behind the use of DS and a few studies found some reasons for taking supplements that include: reducing the risk of dietary deficiency; losing or gaining weight; muscle building; overall wellness to boost immunity and decrease susceptibility to diseases and as an energy source^(2,3)^. Adolescents think they need extra nutrients from DS to compensate for their energy loss for studies and extra-academic activities^(4)^. These supplements are neither meant to cure diseases or health conditions nor to be used as medicines^(5)^.

Researchers reported that approximately one-third of US children and adolescents (34·0 %) take DS^(6)^. In Japan, only 6·8 % of elementary school children were spotted as DS users, and this use was significantly associated with the highest frequency of sports participation, household income and maternal education^(7)^. Another Korean study identified that 33·4 % of the children and adolescents in their study population took DS and concluded that DS were higher in the socio-economically stable groups with good health behaviour^(8)^. A study conducted in Poland reported that about 29·6 % of participants used DS, where gender, residential area, BMI and health status were the predictors of DS use^(9)^. As reported earlier, about 20·1 % of adolescents use DS in the Australian population^(10)^. The previous literature shows that most multivitamins, vitamin D, *n*-3-fatty acids and minerals are the prioritised DS. On the other hand, herbs, probiotics, proteins, fish oils, soya products and botanicals are used less^(6–12)^.

Diversity in demographic characteristics of the population appears to influence the prevalence of supplement use^(9,13)^. A recent study revealed that the DS use was higher among women and older people in Bangladesh. Demographic and lifestyle factors such as monthly income, educational status, sedentary lifestyle and smoking status were reported to correlate with DS use^(14)^. Another survey was conducted in 2018 to investigate the dynamics of DS use and the assessment revealed that approximately 41·3 % of undergraduate female students were DS users according to their previous year’s usage records^(15)^. They use DS for health and well-being, as energy sources, immune boosters and memory enhancers, for weight loss or maintenance and on physician recommendations, among others^(15)^.

The use of DS represents a field of interest because of their potential impact on disease. However, in Bangladesh, surveys on the prevalence and determinants of DS use primarily focus on adults^(4,14,15)^, demonstrating that pregnant and non-pregnant women mainly consume Ca, Zn and Fe supplements, and male and female participants consume DS without prescriptions. At the same time, the number of studies conducted on children and adolescents is limited. Therefore, the main objective of our current study is to evaluate the prevalence of DS and their associated factors among adolescents in the selected parts of Bangladesh.

## Methods

### Study area and settings

A cross-sectional study was conducted in the Kurigram and Patuakhali districts of Bangladesh. The Kurigram district is located in the northern region along with the border of India and is administratively divided into nine Upazilas (sub-districts). Patuakhali district is located in the southern part of Bangladesh and is divided into eight Upazilas.

### Questionnaire and data collection techniques

A structured questionnaire was prepared by critically reviewing relevant literature^(7–10,13–15)^. The survey included socio-demographic characteristics, health-related factors, sources of information on DS, reasons for use and non-use of DS, types of DS used, frequency of DS use, adverse effects of DS use and opinions regarding DS use. Six health professionals and two-BSc qualified nutritionists were recruited for data collection. Data were collected through face-to-face interviews in the schools outside the classroom using a pretested questionnaire. Interviews were conducted only with those participants who agreed to provide the data. Data collection was under the continuous supervision of the authors.

### Sampling and sample size

The study data were collected by a convenient sampling method. Participants were selected based on study purpose (10 to 19 years) with the expectation that each participant would provide unique and rich information to the study. The sample size was determined using the formula for cross-sectional study, 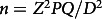 , where *n* is the required sample size, *Z* is 1·96 at a 95 % CI, *D* is the margin of error at 5 % (SD of 0·05) and 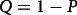 . The value of *P* was considered 50 % because of the unavailability of a similar study among adolescents. Therefore, a minimum sample size of 384 was obtained. There were 734 participants interviewed, of whom 32 were excluded due to missing values, yielding a final analytical sample size of 702 (360 from Patuakhali and 342 from Kurigram). This made the response rate 95·60 %.

### Dependent variable and predictors

**Fig. 1:**
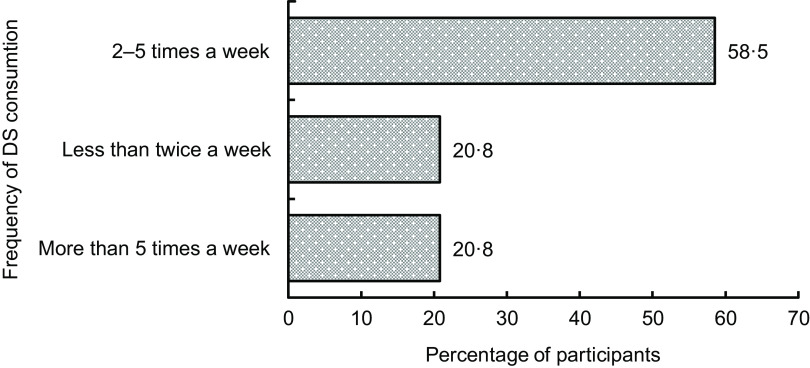
Frequency of dietary supplements consumption (*n* 583)

The dependent variable DS use was categorised as ‘yes’ if the participants had used it in the past month and ‘no’ if they had not used DS in the past month. In addition, socio-demographic and socio-economic variables (age, sex, education, mother’s education, area, income, religion and number of siblings) and other related variables (self-related health status, chronic disease, physical activity, number of meals taken/day, impression about DS for health and knowledge source of DS) were considered as predictors (see Fig. 1 for details).

### Statistical analysis

All the statistical analyses were done using IBM SPSS statistics (version 26.0) and R (version 3.8.1). This study used Microsoft Excel as a calculator and second analysing software. First, simple descriptive tests (Chi-square and Fisher’s exact tests) were done to observe the frequencies, percentages, means and other information regarding variables. Then multivariate logistic regression analysis was used to evaluate the association between DS use and independent variables. The logistic regression model was validated by the ‘Omnibus tests of model coefficient’ and the ‘Hosmer and Lemeshow test’ with a *P*-value of <0·001 (30 df, *χ* ^2^ = 239·18) and 0·116 (517 df, *χ* ^2^ = 555·61), respectively. AOR with 95 % CI and *P* < 0·05 were used to assess the association between dependent and independent variables. *P* < 0·05 were considered significant. A forest plot was used for the graphical display of the significant findings.

## Results

### Demographic and health-related information of the participants

Table 1 represents the socio-demographic and health-related information of 702 participants, of whom 83 % (583) were DS users and 17 % (119) were non-users. The number of female participants was 54·1 % (*n* 380) which was relatively higher than males. Most of the participants (81·8 %, *n* 574) were 15 to 19 years old. In addition, the majority of the participants were from high school (94·6 %, *n* 664). Almost all the participants (88·9 %, *n* 624) lived in rural areas. Furthermore, most of the participants (*n* 429, 61·1 %) had a lower family income level of <15 000 BDT (15 000 BDT = 157·90 US $). Finally, 82·2 % (*n* 577) of participants reported good health status and 88·7 % (*n* 623) of participants were free from chronic diseases.

**Table 1:** Association of demographic and health-related variables with the use of dietary supplements (DS)

Variables	Categories			Dietary supplement				*χ* ^2^	*P*-value
		Total (*n* 702)		DS (-) = 119		DS (+) = 583			
		*n*	%	*n*	%	*n*	%		
Age	10 to 14	128	18·2	27	21·1	101	78·9	1·91	0·167
	15 to 19	574	81·8	92	16·0	482	84·0		
Gender	Male	322	45·9	52	16·1	270	83·9	0·27	0·602
	Female	380	54·1	67	17·6	313	82·4		
Education	High school	664	94·6	107	16·1	557	83·9	6·11	0·013
	College	38	5·4	12	31·6	26	68·4		
Mother’s education	No formal education	25	3·6	7	28·0	18	72·0	6·80	0·147
	Primary school	222	31·6	29	13·1	193	86·9		
	Secondary school	317	45·2	60	18·9	257	81·1		
	Higher secondary school	128	18·2	20	15·6	108	84·4		
	Honor’s and above	10	1·4	3	30·0	7	70·0		
Family income (BDT)	Low (<15 000)	429	61·1	77	17·9	352	82·1	1·63	0·443
	Middle (15 000–30 000)	234	33·3	34	14·5	200	85·5		
	High (>30 000)	39	5·6	8	20·5	31	79·5		
Religion	Muslim	604	86·0	99	16·4	505	83·6	0·97	0·326
	Hindu	98	14·0	20	20·4	78	79·6		
Residence	Urban	78	11·1	11	14·1	67	85·9	0·51	0·477
	Rural	624	88·9	108	17·3	516	82·7		
Number of siblings	1 to 2	301	42·9	53	17·6	248	82·4	25·94	<0·001
	3 to 5	377	53·7	53	14·1	324	85·9		
	More than 5	24	3·4	13	54·2	11	45·8		
Self-reported health status	Poor or fair	20	2·8	8	40·0	12	60·0	24·63	<0·001
	Good	577	82·2	90	15·6	487	84·4		
	Very good	88	12·5	12	13·6	76	86·4		
	Excellent	17	2·4	9	52·9	8	47·1		
Physical ability	Low	24	3·4	8	33·3	16	66·7	5·96	0·051
	Moderate	638	90·9	107	16·8	531	83·2		
	High	40	5·7	4	10·0	36	90·0		
Chronic diseases	No	623	88·7	86	13·8	537	86·2	38·96	<0·001
	Yes	79	11·3	33	41·8	46	58·2		
Number of meals/d	≤3	413	58·8	87	21·1	326	78·9	12·06	0·002
	Four	261	37·2	29	11·1	232	88·9		
	≥5	28	4·0	3	10·7	25	89·3		
DS are good for health	No	30	4·3	16	53·3	14	46·7	137·53	<0·001
	Yes	476	67·8	27	5·7	449	94·3		
	I do not know	196	27·9	76	38·8	120	61·2		
Encourage the use of DS	Yes, I always encourage	239	34·0	17	7·1	222	92·9	163·59	<0·001
	Yes, only on doctor’s recommendation	283	40·3	16	5·7	267	94·3		
	Not at all	180	25·6	86	47·8	94	52·2		
Knowledge sources	Healthcare provider	155	22·1	14	9·0	141	91·0	11·06	0·050
	Internet	99	14·1	21	21·2	78	78·8		
	Professional literature	41	5·8	6	14·6	35	85·4		
	Friends, family members and relatives	241	34·3	43	17·8	198	82·2		
	Social media or seminar	75	10·7	14	18·7	61	81·3		
	Book	91	13·0	21	23·1	70	76·9		

Note: (15 000 BDT = 157·90 US $). DS (−) = Dietary supplement non-users, and DS (+) = Dietary supplement users.

### Sources of knowledge of dietary supplements

The knowledge sources are also explained in Table 1. A major portion of the participants (34·3 %) knew about DS from their friends, family members and relatives. 22·1 % were informed by their healthcare provider. About 14·1 % and 13 % of the participants reported that they learned about DS from the internet and books, respectively.

### Association of demographic and health-related variables with dietary supplement use

The interrelation of demographic and health-related variables with DS use is displayed in Table 1. Education, number of siblings, self-reported health status, chronic diseases, number of meals/d, DS are good for health and encouragement of DS use were significantly associated with DS use.

### Frequency of dietary supplement use

Figure 1 illustrates that 58·5 % of participants used DS 2–5 times a week, 20·8 % used DS less than twice a week and 20·8 % used DS more than 5 times a week (*n* 583).

### Reasons for using and not-using dietary supplements

**Fig. 2:**
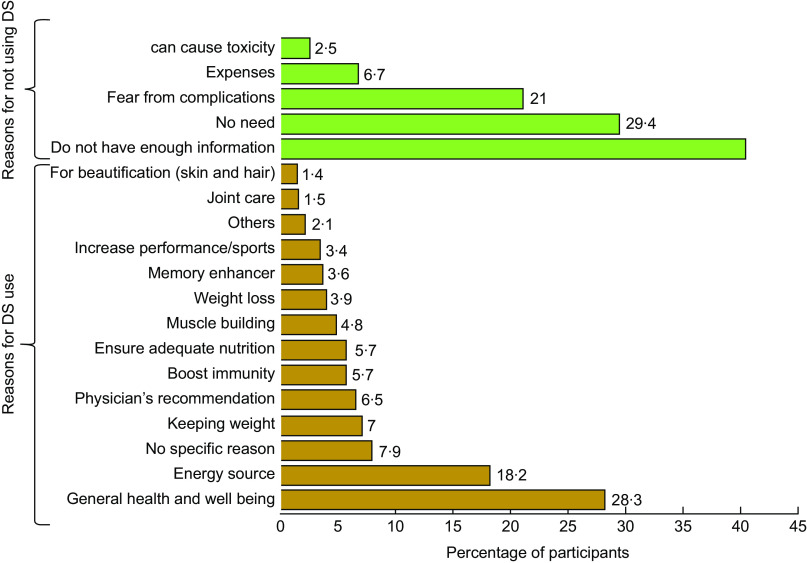
Reasons for using and not using dietary supplements (*n* 583 for DS users and *n* 119 for non-users)

The comprehensive rationales regarding using and non-using participants of DS are represented in Fig. 2 (*n* 583 for DS users and *n* 119 for non-users). A large number of the participants took DS to improve their general health and well-being (28·3 %). A smaller portion of the participants used DS as an energy source (18·2 %). On the other hand, several reasons came out from the survey for not using DS. A greater portion (40·3 %) mentioned that they did not know enough about the use of DS. Moreover, 29·4 % of participants believed that they did not need any DS. Of note, 21 % of the participants did not take supplements due to the fear of complications.

### Types of dietary supplement use

**Fig. 3:**
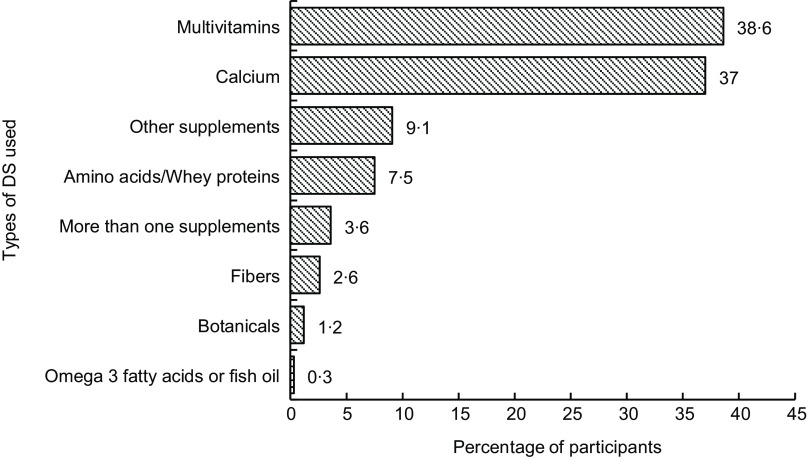
Types of dietary supplements used (*n* 583)

There was a mixed response to the use of supplements among participants (*n* 583). Figure 3 shows that the majority (38·6 %) of supplement users commonly used multivitamins. The second peak of users (37 %) took Ca as DS. Apart from those, 9·1 % of participants reported using other supplements that were not specified.

### Adverse effects of dietary supplement use

**Fig. 4:**
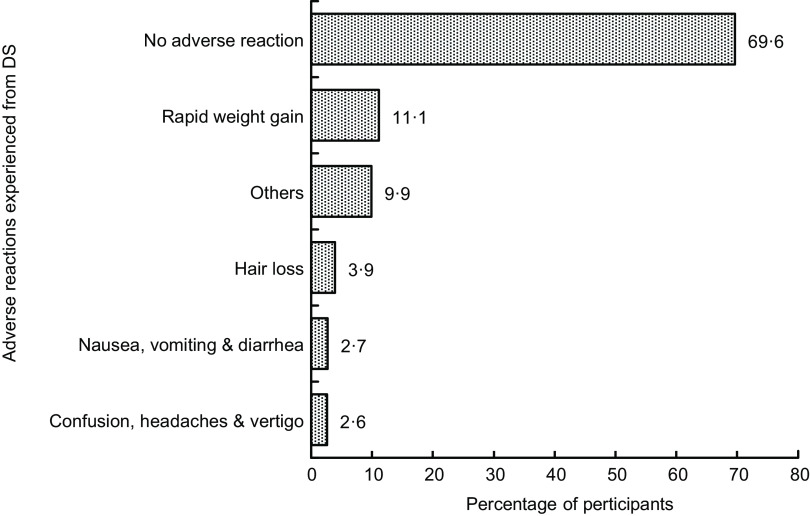
Adverse reactions experienced from dietary supplements (*n* 583)

The interviewers asked participants if they experienced any adverse reactions due to DS use (*n* 583). The results are represented in Fig. 4, where more than half (69·6 %) of users did not face any effect on using DS. In contrast, many of them revealed several adverse effects, which include rapid weight gain (11·1 %), hair loss (3·9 %), nausea, vomiting and diarrhoea (2·7 %), confusion, headaches and vertigo (2·6 %). Excluding these reactions, an observable number of other consequences were encountered by 9·9 % of participants, which were not specified.

### Opinions regarding dietary supplement use

**Fig. 5:**
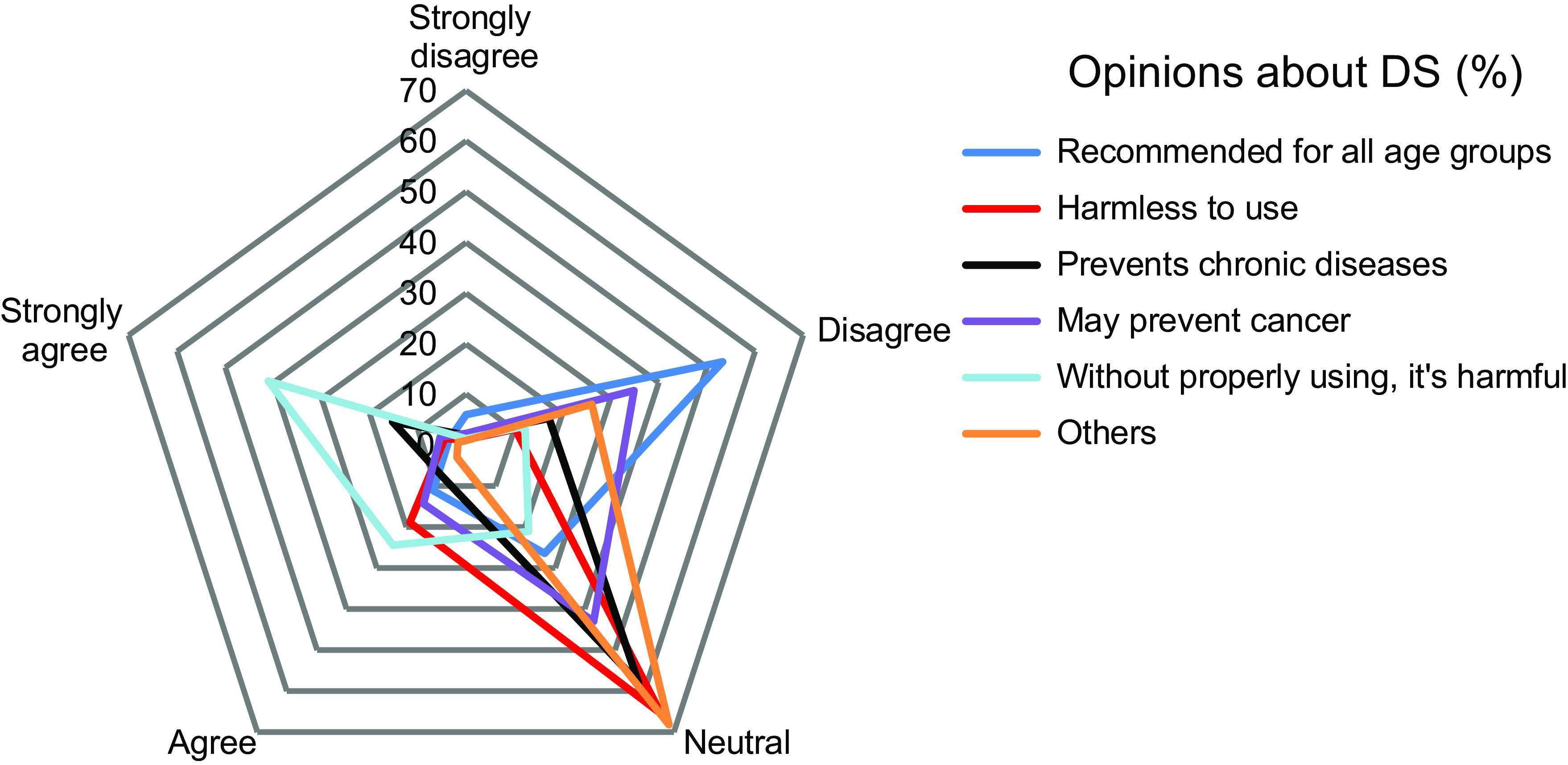
Opinions regarding the use of dietary supplements (*n* 702)

The details of opinions about the use of DS are presented in Fig. 5 (*n* 702). More than 53·3 % of the adolescents disagreed with recommending DS for all age groups, whereas 65·5 % of the participants were neutral that DS use seemed harmless. Besides, approximately 60 % of participants showed neutral thoughts that the use of DS might prevent chronic diseases. Over 40 % of the participants were neutral about whether DS might prevent cancer. On the contrary, 30 % of participants disagreed with this opinion. Another opinion came forward from the participants that if DS were improperly used, they might be harmful to health and 41 % of participants strongly agreed with this opinion. The rest of the participants had non-specified explanations, most of which were neutral (approximately 70 %).

### Predictors of dietary supplement use

**Fig. 6:**
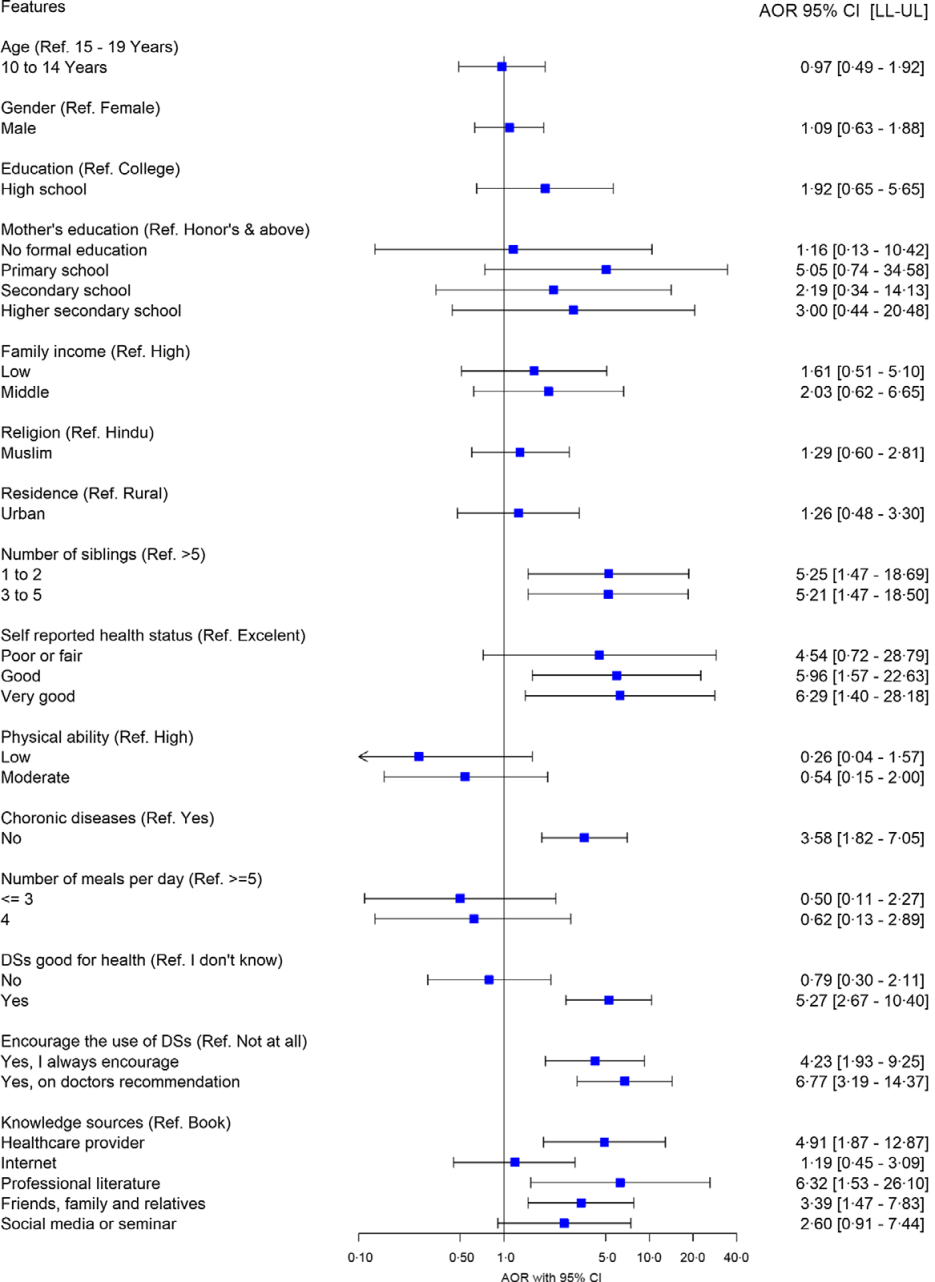
Forest plot of OR and 95 % CI representing the correlation between predictors and dietary supplement use

Figure 6 puts on a view of predictors of DS use. DS use was associated with the number of siblings, self-reported health status, chronic disease, perceived health benefits of DS, encouragement of DS use and knowledge sources (a resource where information about DS can be found). Participants with ≤2 siblings (AOR = 5·25, 95 % CI (1·47, 18·69)) and those with 3 to 5 siblings had higher odds (AOR = 5·21, 95 % CI (1·47, 18·50)) than those with ≥5 siblings. In terms of self-reported health status, participants with good health status (AOR = 5·96, 95 % CI (1·57, 22·63)) and very good health status had higher odds (AOR = 6·29, 95 % CI (1·40, 28·18)) than those with excellent health status. Participants who did not have chronic disease had higher odds (AOR = 3·58, 95 % CI (1·82, 7·05)) than those who did. Participants who claimed DS were good for their health had higher odds (AOR = 5·27, 95 % CI (2·67, 10·40)) than those who did not know whether DS were good for their health. Participants who always encouraged others (AOR = 4·23, 95 % CI (1·93, 9·25)) and encouraged others on the doctor’s advice had higher odds (AOR = 6·77, 95 % CI (3·19, 14·37)) than those who never encouraged others. Participants whose knowledge source was healthcare providers had higher odds (AOR = 4·91, 95 % CI (1·87, 12·87)) than those whose knowledge source was the book. Moreover, participants whose knowledge source was professional literature (AOR = 6·32, 95 % CI (1·53, 26·10)), and friends, family and relatives had greater odds (AOR = 3·39, 95 % CI (1·47, 7·83)) in contrast to those who knew by the book.

## Discussion

The current cross-sectional study was conducted to demonstrate the prevalence of DS use and the related factors among adolescents in Bangladesh. This survey was specifically carried out with male and female teenagers from 10 to 19 years of age. The demographic and health characteristics greatly influenced the prevalence of DS use among adolescents. The prevalence of DS use in Bangladeshi adolescents (83 %) seemed higher than in Bangladeshi adults (41 %), Bangladeshi undergraduate female students^(14,15)^, Japanese adolescents (20·4 %)^(16)^, Australian teenagers (20·1 %)^(10)^, Canadian adolescents (42·5 %)^(17)^ and Korean children and adolescents (34 %)^(8)^. However, there was no data available for Bangladeshi adolescents to compare with. The higher prevalence of DS use in this study compared to Bangladeshi adults and adolescents from other countries might be due to the increased availability and wide variety of DS across the country, such as in pharmacies and drug and DS markets, as the sale of supplements is not regulated in Bangladesh^(15)^. There is no regulation and health policy on the production and use of DS in Bangladesh compared to countries (including Canada, Australia, Japan and Korea) applying regulations to DS considerably^(8,10,16,17)^. DS use in Bangladesh might also be influenced by commercial advertisements.

Across all participants in our study, the most commonly reported type of DS used was multivitamins, which is in line with other literature^(18,19)^. Ca was the most supplemented mineral, similar to the results of a German study^(20)^. The higher use of multivitamins and Ca in this study might be due to their availability in pharmacies, large discount stores and online.

The adolescents were asked about the reasons behind the use of DS. In accordance with previous studies^(18,19)^, the main reason for using DS was to improve or maintain general health and well-being, followed by energy sources. Moreover, we found that only a few took those supplements on a physician’s recommendation, while the majority did not even realise they needed a recommendation and willingly used supplements on their own. Some participants had no particular reason as they were unsure about their use. It may indicate a lack of knowledge that leads them to misinformation and uncertainty. Thus, the results of this study contribute to a deeper understanding of the reasons for dietary supplementation by adolescents.

This current study reported several adverse effects of taking DS, although the majority of the participants reported no adverse effects, similar to other studies^(15,21)^. However, when taken in excess, DS may have adverse effects^(22,23)^. Interestingly, similar to a previous study, a large proportion of the participants strongly agreed that DS are harmful if not used properly^(24)^. Surprisingly, another recent study provides results that are incompatible with the findings of our study^(15)^, maintains that DS are safe to use and ignores the necessity for paying attention to the use of DS.

In this study, we observed that a small number of siblings was associated with a higher intake of DS, whereas the other studies found no such association^(15,25)^. One prominent explanation is that a small number of siblings might increase personal finances and household savings, thereby enhancing the use of DS.

Similar to recent studies^(7,13)^, an interesting finding was that DS use was more prevalent among adolescents with good and very good health status. These findings are unique and require further elucidation. In our study, adolescents suffering from chronic diseases consume fewer DS might reflect that the DS used in Bangladeshi adolescents are mainly for health promotion but not for the treatment of diseases^(8,9)^.

In agreement with extant literature, most of the participants from the current study believed that DS are good for health and used more DS while a negligible number of the participants disagreed with the statement^(15,26)^. Moreover, the largest portion of the participants recommended DS only on a doctor’s recommendation and used more DS. Although, all the given frequencies of the participants regarding other statements are almost similar. According to our study, knowledge sources have an impact on DS use, and it shows that gathering knowledge from healthcare professionals, family and friends and professional literature contributes to higher DS use. This could be explained by the fact that participants from the study area were influenced by their family and friends and by healthcare professionals while practicing DS.

The major limitation of this study was that it was carried out in a few districts in limited areas. Hence, our results are not generalisable. In addition, we did not examine the dosage used for the DS nor the duration of use by the participants. Information about nutritional status-related parameters was not available. Since our data were cross-sectional, no causal inferences can be drawn. Despite this point, we believe that our study will be a great resource of research information to understand the current state of DS use among adolescents and build an ideal direction in the case of future supplementary uses. Furthermore, adolescents and parents may understand the importance of the involvement of health professionals and authorities before planning to take such DS to avoid harmful effects.

## Conclusions

In summary, the present findings are that 83 % of Bangladeshi adolescents use DS, a higher prevalence than what has been found in adolescents living in other countries. Many of these participants believe that DS are good for their general health based on information obtained from Friends, family members and relatives, healthcare providers and the internet, but a significant number may not recognise the potential for adverse effects. Therefore, healthcare professionals should assess whether or not their young patients use DS and ensure that they are appropriately informed about these supplements’ potential benefits and risks. In addition, further research is needed, encompassing a wide variety of DS, including a larger study population, exploring nutritional supplementations along with nutritional status, food habits and health.
